# Supplementary material for: Exploring the Relationship between (De-)Centralized Health Care Delivery, Client-Centeredness, and Health Outcomes—Results of a Retrospective, Single-Center Study of Transgender People Undergoing Vaginoplasty
Source: Healthcare (Basel). 2023 Jun 14;11(12):1746. doi: 10.3390/healthcare11121746 (PMC10297987; doi:10.3390/healthcare11121746)
Supplement: Supplementary file 1 [file healthcare-11-01746-s001.zip › healthcare-2349829-supplementary.pdf]

STROBE Statement—Checklist of items that should be included in reports of ***cross-sectional studies***

	Item No	Recommendation
**Title and abstract**	1	(*a*) Indicate the study's design with a commonly used term in the title or the abstract<br>*The title describes the study design as "a retrospective, single-center study".*
		(*b*) Provide in the abstract an informative and balanced summary of what was done and what was found<br>*The abstract describes the methods and findings.*
**Introduction**		
Background/rationale	2	Explain the scientific background and rationale for the investigation being reported<br>*The background and rationale are described in the Introduction, paragraphs 1,2,3 & 4.*
Objectives	3	State specific objectives, including any prespecified hypotheses<br>*The specific aims of the study are stated in the Introduction, paragraph 5.*
**Methods**		
Study design	4	Present key elements of study design early in the paper<br>*The study design is discussed in the subsection "Study design" of the Methods section.*
Setting	5	Describe the setting, locations, and relevant dates, including periods of recruitment, exposure, follow-up, and data collection<br>*The institutional setting and study location are described in the subsection "Study design" of the Method section.*
Participants	6	(*a*) Give the eligibility criteria, and the sources and methods of selection of participants<br>*Selection of the sample is discussed in the subsections "Participants" and "Participant recruitment" of the Methods section.*
Variables	7	Clearly define all outcomes, exposures, predictors, potential confounders, and effect modifiers. Give diagnostic criteria, if applicable<br>*All variables assessed in the study are discussed in the "Measures" subsection and the supplementary material.*
Data sources/ measurement	8*	For each variable of interest, give sources of data and details of methods of assessment (measurement). Describe comparability of assessment methods if there is more than one group<br>*Measurement of the variables are discussed in the "Measures" subsection and the supplementary material.*
Bias	9	Describe any efforts to address potential sources of bias<br>*Potential bias is discussed in the last paragraph of the "Participant recruitment" subsection of the Methods section.*
Study size	10	Explain how the study size was arrived at<br>*Sample determination is discussed in the subsections "Participants" and "Participant recruitment" of the Methods section.*
Quantitative variables	11	Explain how quantitative variables were handled in the analyses. If applicable, describe which groupings were chosen and why<br>*Use of variables is discussed in the "Measures" and the "Data Analysis" subsection of the Methods section.*
Statistical methods	12	(*a*) Describe all statistical methods, including those used to control for confounding<br>*Statistical methods are discussed in the "Data Analysis" subsection.*

		(*b*) Describe any methods used to examine subgroups and interactions<br>*Described in the "Data analysis" subsection.*
		(*c*) Explain how missing data were addressed<br>*Missing observations are discussed in the "Data analysis" subsection.*
		(*d*) If applicable, describe analytical methods taking account of sampling strategy<br>*None.*
		(*e*) Describe any sensitivity analyses<br>*None.*
**Results**		
Participants	13*	(a) Report numbers of individuals at each stage of study—eg numbers potentially eligible, examined for eligibility, confirmed eligible, included in the study, completing follow-up, and analysed<br>*Data collection completion rates are discussed in the subsections "Participants" and "Participant recruitment" of the Methods section.*
		(b) Give reasons for non-participation at each stage<br>*Discussed in the subsection "Participant recruitment" of the Methods section.*
		(c) Consider use of a flow diagram<br>*No STROBE flowchart was prepared, as the inclusion and exclusion of participants was already discussed in the subsections "Participants" and "Participant recruitment" of the Methods section.*
Descriptive data	14*	(a) Give characteristics of study participants (eg demographic, clinical, social) and information on exposures and potential confounders<br>*Participants characteristics are presented in Table 1.*
		(b) Indicate number of participants with missing data for each variable of interest<br>*Can be assessed for the individual variable on the basis of the data from tables 1,2,3,4, & 5*
Outcome data	15*	Report numbers of outcome events or summary measures<br>*Both numbers and percentages/proportions are reported throughout the Results Section.*
Main results	16	(*a*) Give unadjusted estimates and, if applicable, confounder-adjusted estimates and their precision (eg, 95% confidence interval). Make clear which confounders were adjusted for and why they were included<br>*Unadjusted results are presented for all outcomes.*
		(*b*) Report category boundaries when continuous variables were categorized<br>*Not applicable.*
		(*c*) If relevant, consider translating estimates of relative risk into absolute risk for a meaningful time period<br>*Not applicable.*
Other analyses	17	Report other analyses done—eg analyses of subgroups and interactions, and sensitivity analyses<br>*Hierarchical cluster analyses are reported in the Results section.*
**Discussion**		
Key results	18	Summarise key results with reference to study objectives<br>*Results are summarized in paragraph "Main findings" of the Discussion section.*
Limitations	19	Discuss limitations of the study, taking into account sources of potential bias or imprecision. Discuss both direction and magnitude of any potential bias<br>*Limitations are discussed in last paragraph of the Discussion section "Limitations of*

		this study".
Interpretation	20	Give a cautious overall interpretation of results considering objectives, limitations, multiplicity of analyses, results from similar studies, and other relevant evidence<br>*Paragraph "What is already known on this topic and what this study adds" and "Limitations of this study" of Discussion section.*
Generalisability	21	Discuss the generalisability (external validity) of the study results<br>*Paragraph "What is already known on this topic and what this study adds" and "Limitations of this study" of Discussion section.*
**Other information**		
Funding	22	Give the source of funding and the role of the funders for the present study and, if applicable, for the original study on which the present article is based<br>*"Funding" section at the end of the manusript.*

*Give information separately for exposed and unexposed groups.

Note: An Explanation and Elaboration article discusses each checklist item and gives methodological background and published examples of transparent reporting. The STROBE checklist is best used in conjunction with this article (freely available on the Web sites of PLoS Medicine at http://www.plosmedicine.org/, Annals of Internal Medicine at http://www.annals.org/, and Epidemiology at http://www.epidem.com/). Information on the STROBE Initiative is available at www.strobe-statement.org.
